# Community composition of arbuscular mycorrhizal fungi associated with native plants growing in a petroleum‐polluted soil of the Amazon region of Ecuador

**DOI:** 10.1002/mbo3.703

**Published:** 2018-08-16

**Authors:** Mónica Garcés‐Ruiz, Carolina Senés‐Guerrero, Stéphane Declerck, Sylvie Cranenbrouck

**Affiliations:** ^1^ Laboratory of Mycology Earth and Life Institute Université catholique de Louvain Louvain‐la‐Neuve Belgium; ^2^ Laboratorio de micología Carrera de Microbiología, Facultad de Ciencia Exactas y Naturales Pontificia Universidad Católica del Ecuador Quito Ecuador; ^3^ Tecnologico de Monterrey Nuevo México Zapopan México; ^4^ Laboratory of Mycology Mycothèque de l'Université catholique de Louvain (MUCL/BCCM) Earth and Life Institute, Université catholique de Louvain Louvain‐la‐Neuve Belgium

**Keywords:** amazonian soil, Arbuscular mycorrhizal fungi, Ecuador, 454‐pyrosequencing, community composition, hydrocarbon ‐ polluted environment

## Abstract

Arbuscular mycorrhizal fungi (AMF) are worldwide distributed plant symbionts. However, their occurrence in hydrocarbon‐polluted environments is less investigated, although specific communities may be present with possible interest for remediation strategies. Here, we investigated the AMF community composition associated with the roots of diverse plant species naturally recolonizing a weathered crude oil pond in the Amazon region of Ecuador. Next generation 454 GS‐Junior sequencing of an 800 bp LSU rRNA gene PCR amplicon was used. PCR amplicons were affiliated to a maximum‐likelihood phylogenetic tree computed from 1.5 kb AMF reference sequences. A high throughput phylogenetic annotation approach, using an evolutionary placement algorithm (EPA) allowed the characterization of sequences to the species level. Fifteen species were detected. *Acaulospora* species were identified as dominant colonizers, with 73% of relative read abundance, *Archaeospora* (19.6%) and several genera from the Glomeraceae (*Rhizophagus, Glomus macrocarpum‐*like*, Sclerocystis, Dominikia* and *Kamienskia*) were also detected. Although, a diverse community belonging to Glomeraceae was revealed, they represented <10% of the relative abundance in the Pond. Seventy five % of the species could not be identified, suggesting possible new species associated with roots of plants under highly hydrocarbon‐polluted conditions.

## 1 INTRODUCTION

Oil pollution is a current problem whose effects on fauna and flora are perceptible in natural as well as anthropogenic environments (Atlas & Philp, 2005; Labana, Kapur, Malik, Prakash, & Jain, 2007). It is often reported that contamination by hydrocarbons changes the microbial community structure and decreases the microbial diversity (Satyanarayana, Johri, & Prakash, 2012). However, some microbial populations have demonstrated their capacity to adapt to the pollutants, resulting in the development of microbial consortia able to degrade a variety of petroleum molecules (Sahoo, Ramesh, & Pakshirajan, 2012). Due to the fast‐growing demand for hydrocarbon derivatives all over the world (Lee, 2015), it is expected that environmental pollution will increase in the coming years (Öztürk et al., 2015). It is thus of the highest priority either to apply physicochemical or to develop biological‐friendly remediation strategies. The identification of native microbial communities well‐adapted to polluted conditions and their further isolation, mass production and application to polluted soils are part of this strategy and may represent an interesting approach for handling oil‐contaminated sites.

The Amazonian region of Ecuador is a hotspot of biodiversity, which unfortunately is also a major reservoir of hydrocarbons (Ministerio del Ambiente de Ecuador, 2016). Therefore, the effects of petroleum pollutants on fauna and flora regularly reported in the literature (see above) are also significant here. Nevertheless, in hydrocarbon‐polluted sites of the Charapa field in the Amazonian region, a natural recolonization of the abandoned weathered oil ponds was observed through the years (Garcés‐Ruiz, Senés‐Guerrero, Declerck, & Cranenbrouck, 2017). This suggested that plant roots and microbial communities associated with the rhizosphere were able to establish, probably enhancing the degradation of petroleum compounds, and thus representing a potentially important approach for the in situ treatment of hydrocarbon‐polluted soils (Öztürk et al., 2015).

Among the rhizosphere microbial communities, one of the most important is the arbuscular mycorrhizal fungi (AMF). These obligate root symbionts contribute to the formation and stability of soil aggregates, and to the transport of nutrients and water to the plants (Smith & Read, 2008). Thus, phytoremediation assisted by AMF has been suggested for hydrocarbon‐polluted environments (Lenoir, Lounes‐Hadj Sahraoui, & Fontaine, 2016). The application of AMF may enhance plant growth and nutrient uptake. Several studies, in controlled conditions, reported an increased plant biomass, root and shoot length, P and N uptake and chlorophyll content (Liu & Dalpé, 2009; Tang, Chen, Huang, & Tian, 2009; Wu, Yu, Wu, Lin, & Wong, 2011). An increase in biodegradation activity of roots and rhizosphere microorganisms was also demonstrated, as well as an improved absorption and bioaccumulation of hydrocarbons by roots (see review Rajtor & Piotrowska‐Seget, 2016). A recent study, conducted in a hydrocarbon‐polluted soil from a natural environment in the Amazonian region of Ecuador (i.e., the Charapa field, Garcés‐Ruiz et al. (2017)) recorded the presence of a relatively diverse community of AMF associated with various herbaceous plants. A high root colonization was noticed in all the plants sampled and a molecular diversity analysis, using a clone library and Sanger sequencing approach of a 1.5 kb fragment defined as the DNA barcode for AMF (Stockinger, Krüger, & Schüßler, 2010) allowed the identification of four AMF genera (i.e., *Glomus, Rhizophagus, Acaulospora* and *Archaeospora*) associated with three plant species (*Euterpe precatoria*,* Carludovica palmata* and *Costus scaber*) (Garcés‐Ruiz et al., 2017). However, more than 74% of the species could not be ascribed to an identified AMF, suggesting the presence of numerous unidentified taxa.

The objective of this study was to explore in‐deep the community composition of AMF associated with roots of a diverse assemblage of plants present in a weathered crude oil Pond, from the Charapa field (see Garcés‐Ruiz et al., 2017), which could possibly be used to assist in phytoremediation efforts. High‐throughput 454‐pyrosequencing of an ~800 bp rDNA fragment was conducted and analyzed, using a reference “phylogenetic backbone” based on long AMF sequences (i.e., SSU‐ITS‐LSU 1.5 kb fragment) (Krüger, Krüger, Walker, Stockinger, & Schüßler, 2012; Stockinger et al., 2010) and an evolutionary placement algorithm (EPA), which allows the phylogenetic annotation of sequences to the species level (Senés‐Guerrero & Schüßler, 2016a). The AMF community composition in plant roots was evaluated according to the site of collection within the Pond by comparing the relative read abundance (RA) of AMF species (Loján et al., 2017; Senés‐Guerrero & Schüßler, 2016a).

## 2 MATERIALS AND METHODS

### 2.1 Sampling location and experimental design

**Figure 1:**
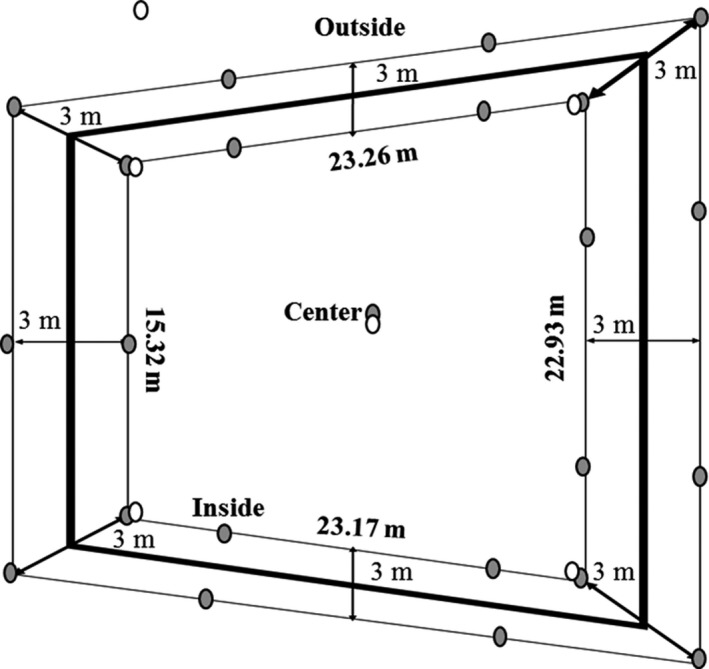
Schematic representation of the weathered oil‐pond in the Charapa field. Sampling points of plants (gray circles) and soil (white circles), outside, inside and center. Not at scale

The sampling was done on December 2013 in a weathered crude oil Pond of 450 m^2^ in the Charapa field (76°48′54″ W, 00°11′46″ S) located in the province of Sucumbíos in the Amazonian region of Ecuador. More details and description of this site can be found in Garcés‐Ruiz et al. (2017). The Pond has an irregular shape; the west, north, and south sides measured around ~23 m while the east side was only ~15 m. The perimeter of the Pond was marked every ~ 7.3 m (11 points in total). From each point, a straight line to the center of the Pond was traced and plants were sampled 3 m inside and 3 m outside the Pond. Due to the complexity of the sampling environment, only one extra point was selected close to the center of the Pond (Figure 1). In total, 40 plants were collected (18 inside and outside and 4 in the center) (Table 1). The number of plants sampled at each point varied from 1 to 3 according to their abundance and in a few cases, no plant was present.

**Table 1:** Plant species and number of individuals collected outside, inside and in the center of the pond

Plant species	3 m outside	3 m inside	Center
*Piper* sp.	1	2	
*Miconia* sp.	1		
*Inga auristellae*	2		
*Costus* sp.	1	3	
*Calathea*		1	
*Anthurium* sp.	1	2	
*Heliconia* sp.	1		
*Euterpe precatoria*	1	2	
*Urera baccifera*	1	1	
*Urera caracasana*		1	
*Stigmatopteris*		1	
*Acalypha*	1		
*Geonoma macrostachys*		1	
*Poaceae*	2		2
*Pouruma* sp.	1		
*Leandra* sp.	1		
*Cyathea* sp.	1	1	
*Dendropanax* sp.	1		
*Caladium* sp.		1	
*Cordia*		1	
*Acacia*	1		
*Renealmia* sp.	1		
*Cyclanthus bipartitus*		1	
*Faramea* sp.			1
*Paullinia* sp.			1
Total	18	18	4

### 2.2 Root processing

After sampling, the shoots were used for plant identification and separated from roots that were kept within the soil at 4°C. Roots were cleaned from the soil particles with tap water. They were further washed for 10 min with Tween 80 (Panreac, Spain) diluted in sterilized distilled water (3%), to detach the crude oil (from Gordillo and Decock, personal communication). Roots were finally rinsed with sterilized distilled water. The cleaned roots were divided for evaluation of AMF colonization and DNA extraction.

### 2.3 Physicochemical soil analysis

The Pond consisted of a layer of organic matter and soil above the weathered crude oil known as “tar”. Soil was sampled below the organic matter and until 30 cm depth, at different places inside the Pond. Three independent samples and two composite samples, each of 500 g, were analyzed: (1) a surface sample and (2) a sample collected at 30 cm depth, close to the center of the pond, (3) a composite surface sample and (4) a composite sample collected at 30 cm depth, made of soil cores collected in the 4 corners inside the pond, and (5) a sample at 30 cm depth collected 70 m outside the Pond (i.e., nonpolluted soil – as control). The physicochemical analyses were conducted by the CESAQ‐PUCE laboratory (Table 2). The reference methods used were from the Environmental Protection Agency (US.EPA) and the Standard Methods for the Examination of Water and Wastewater (SM). The analysis performed were pH (US.EPA 9045 D, 2004), organic matter percentage (gravimetric), P mg Kg^−1^ (SM 4500 P, 1999), N mg Kg^−1^ (SM 4500‐N, 1997), K mg Kg^−1^ (EPA 3051/7000A) and total petroleum hydrocarbon (TPH) mg Kg^−1^ (SM 5520 E; US.EPA 3550 B, 2012).

**Table 2:** Chemical and physical analysis from soil collected in Pond in the Charapa field

Analysis	Sample 1	Sample 2	Sample 3	Sample 4	Sample 5 (control)	Analytical method
pH	8	6	5	5	8.1	CP‐PEE‐S004
Organic matter %	>95	>95	>95	>95	44.55	GRAVIMETRIC
P mg Kg^−1^	>450	404.8	>450	321.8	49	SM 4500 P B‐C
N mg Kg^−1^	>1500	666.6	>1500	1138.6	>1500	SM 4500‐N C
K mg Kg^−1^	133.7	42.3	246.1	26.8	82.1	EPA 3051/7000A
TPH mg Kg^−1^	>5000	>5000	>5000	>5000	1188.8	CP‐PEE‐S003

Chemical and physical analysis from three independent samples and two composite samples: (1) a surface sample and (2) a sample collected at 30 cm depth, close to the center of the pond, (3) a composite surface sample and (4) a composite sample collected at 30 cm depth, made of soil cores collected in the 4 corners inside the pond, and (5) a sample at 30 cm depth collected 70 m outside the Pond (i.e., nonpolluted soil – as control).

### 2.4 AMF Root colonization

After cleaning, the roots were stained in acidic‐blue ink (Walker, 2005) and the percentage of total colonization (%TC), arbuscular (%AC), and spores/vesicles (%VC) colonization were estimated under a dissecting microscope (Olympus BH2–RFCA, Japan) at 10× magnification following the method of McGonigle, Miller, Evans, Fairchild, and Swan (1990). An approximate of 100 intersections was observed per sample.

### 2.5 DNA extraction

DNA was extracted from the 40 root samples according to Garcés‐Ruiz et al. (2017). In brief, ~70 mg of dried roots from each sample were ground and material was transferred into the Lysing Matrix E tube from the FastDNA SPIN Kit for Soil (MP Biomedicals, USA). DNA was extracted following the manufacturer's protocol. The DNA integrity was visualized in 1% electrophoresis gel, and 5 μl of the product were stained with 100 × GelRed^™^ (Nucleic Acid Gel Stain, Biotium, Belgium). Samples were run at 100 V for 18 min in 0.5× TAE buffer and stored at −20°C until further use.

### 2.6 PCR conditions and 454‐pyrosequencing

Two PCRs were performed. The first PCR was developed according to Krüger, Stockinger, Krüger, and Schüßler (2009). The amplification of the partial SSU, the complete ITS region and partial LSU rRNA gene, using the SSUmAf–LSUmAr or SSUmCf‐ LSUmBr primers pairs was done. The primers targeted a 1.8 or 1.5 kb region, respectively. The nested PCR was performed as described by Senés‐Guerrero and Schüßler (2016b), where the product of the first one served as template. Nested PCR primer pairs amplified a fragment of around 800 bp from the LSU rRNA gene region. Amplicons were amplified, using fusion primers. The forward primer LSU‐D1f (5′‐TAAGCGGAGGAAAAGAAAMTAAC‐3′) was synthesized together with the 454 adaptor A (5′‐CGTATCGCCTCCCTCGCGCCATCAG‐3′) and different multiplex identifiers (MIDs). Three different MIDs were used according to the site where the plants were sampled (i.e., MID 1: 3 m outside, MID 2: 3 m inside and MID 3: center of the Pond). The reverse primer LSUmBr (Krüger et al., 2009) was synthesized with the 454 adaptor B (5′‐CTATGCGCCTTGCCAGCCCGCTCAG‐3′) (Sigma, Germany). The reaction mix contained 0.02 U/μl Phusion polymerase (Thermo Scientific, Lithuania), 1× Phusion HF Buffer with 1.5 mm MgCl_2_, (Thermo Scientific, Lithuania) 200 μM of each dNTP (Promega, USA), 0.2 μg/ml BSA (Albumin Bovine, AMRESCO, USA) and 0.5 μM of each primer (Sigma, Germany) with 5 μl of template DNA in 20 μl of final reaction. Thermal cycling was done in an Eppendorf Mastercycler Gradient (Eppendorf, Germany) with the following conditions for the first PCR: Five minutes initial denaturation at 99°C; 40 cycles of 10 s denaturation at 99°C, 30 s annealing at 60°C and 1 min elongation at 72°C; and a 10 min final elongation.

In the nested PCR, 1 μl of the first PCR product was used in the final reaction (20 μl). The thermal cycling conditions were the same as for the first PCR, except that only 25 cycles were done (Senés‐Guerrero & Schüßler, 2016a). For each sample, three separate PCRs were performed and the products were loaded on 1% agarose gel electrophoresis as above. Then, PCR replicates for each sample were pooled after confirming a visible band. The pooled products were loaded on 1% agarose to purify with the QIAquick^®^ Gel Extraction Kit (Qiagen, Germany). DNA quantification was performed with the Quant‐iTTM PicoGreen dsDNA Assay Kit (Life technologies, USA) following the manufacturer's protocol. The samples were quantified in a fluorimeter (Fluoroskan Ascent FL, Labsystem, USA) with the Ascent Software (Louisc, nsku91). According to the results, the samples were diluted until they reached 10^9^ molecules μL^−1^. The samples were mixed to equimolar concentration according to the labeled MID (i.e., 1, 2 and 3). Finally, diluted PCR products were pooled in an equimolar concentration to obtain only one sample. 454 pyrosequencing was done by using the 2 XLR GS Junior Sequencing (Nucleomics Core, Leuven Belgium, http://www.nucleomics.be/).

### 2.7 Bioinformatic analyses

Analyses were performed according to Senés‐Guerrero and Schüßler (2016a,b). In an initial step, sequences were quality‐filtered and clustered at 98% to obtain one representative sequence (RS) per cluster. The next step involved the phylogenetic placement by EPA of the RS into a reference phylogenetic tree (Figure S1). The QIIME pipeline (Caporaso et al., 2010) was used for the initial analysis. The parameters to select reads for downstream analyses consisted on reads with no more than 15 ambiguous bases, a maximum length of homopolymer run of 15, a maximum number of 5 primer mismatches and sequences with a minimum length of 500 bp including the primers. The remaining sequences were clustered at a 98% similarity threshold to obtain RS and to avoid merging of different species in the same cluster (Senés‐Guerrero & Schüßler, 2016a). After clustering, singletons were removed and the remaining RS were blasted against the NCBI nucleotide database using Blast2GO (Conesa et al., 2005) to identify and remove non‐AMF sequences.

The remaining RS (with no non‐AMF sequences and no singletons) were taken for species delimitation by means of the RAxML EPA with the GTRGAMMA model performed through a web interface (Berger, Krompass, & Stamatakis, 2011; Berger & Stamatakis, 2011) using a “phylogenetic backbone tree” (Figure S1) based on 1.5 kb reference sequences (Krüger et al., 2012) for sequence placement. The branches of the phylogenetic backbone tree show the placement of the short sequences by EPA. To allow comparisons, species were annotated with the same species numbers as used in previous studies (Loján et al., 2017; Senés‐Guerrero & Schüßler, 2016a; Senés‐Guerrero, Torres‐Cortés, Pfeiffer, Rojas, & Schüßler, 2014).

The sequences were deposited at NCBI with accession numbers MH503958 to MH504107.

### 2.8 Statistical and data analysis

Data for AMF root colonization percentage were analyzed by one way ANOVA. Normal distribution was checked and nonnormal data were normalized by arcsine transformation before analysis. One way ANOVA was used to determine significant difference between sites AMF root colonization.

Statistical analyses were performed, using the IBM SPSS statistic 25 software.

To compare between sites the AMF community composition, 454‐read relative abundance and nonmetric multidimensional scaling (NMDS), were the criteria (Senés‐Guerrero & Schüßler, 2016a). Data for NMDS were square root normalized and analyzed, using Bray‐‐Curtis dissimilarities in the vegan package (Oksanen et al., 2017) of R version 3.3.3 (R Development Core Team 2016).

The Shannon diversity index (H′) was calculated using the formula:

$$H^{\prime }=-\sum \text{pi log (pi)}$$

where pi is the proportional abundance of AMF species according to the site of sampling.

## 3 RESULTS

### 3.1 Physicochemical soil properties

The level of total petroleum hydrocarbon (TPH) in the Pond was >5000 mg Kg^−1^ (limit of quantification LOQ), while in the control (sample 5) it was 1188.8 mg Kg^−1^ (Table 1). The pH was alkaline in the control and in the superficial sample from the middle of the pond (sample 1) (Table 2). The pH was neutral in samples 2, 3, and 4 (Table 2). The mineral nutrient content (P, N and K) was higher in the superficial samples (1 and 3), although the sampling was performed below the organic matter layer. Conversely, sample 5 (control) had a low amount of P compared with the other samples (Table 2) while N was similar or higher compared to the others as well as K (Table 2). The analysis of organic matter was higher than the LOQ in all the samples with the exception of the control soil (Table 2).

### 3.2 AMF root colonization

The roots of all the plants sampled contained AMF structures. Colonization percentages (i.e., total (%TC), arbuscular (%AC) and spores/vesicles (%VC) colonization) were analyzed according to the sampling place (outside, inside and in the center of the Pond). The %TC was 62.5% ± 4.3, 51.2% ± 5.1 and 43.8% ± 13.3, outside, inside and in the center of the Pond, respectively, without any significant difference (*p* = 0.138). The %VC outside the Pond was 4.7% ± 0.9 while it was higher inside and in the center of the Pond (i.e., 10.2% ± 1.9 and 11.5% ± 6.2, respectively). However, no significant difference was noticed (*p* = 0. 058). The %AC did not differ between the sampling places (*p* = 0.054). The values were low and varied from 1.05% ± 0.46 to 2.3% ± 1.2, outside and inside the Pond, respectively. No arbuscules were observed in plant roots from the center of the Pond.

### 3.3 AMF community composition of roots

**Figure 2:**
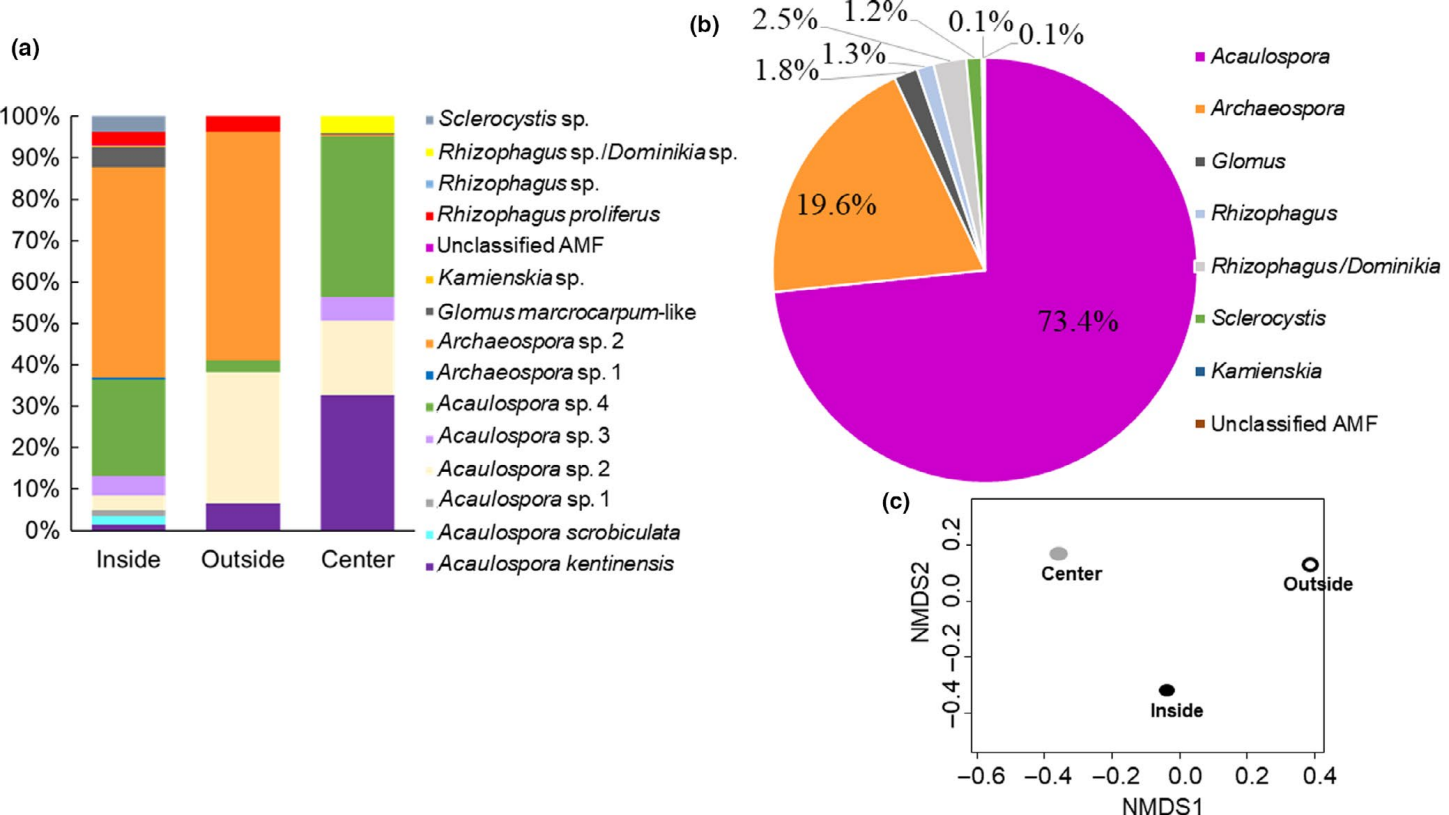
(a) Relative read abundance (%) of Arbuscular mycorrhizal fungi (AMF) species present in pooled root samples from three different sides of the hydrocarbon polluted pond. (b) Pie chart shows the relative abundance (%) of each genus found in the hydrocarbon polluted pond. (c) nonmetric multidimensional scaling (NMDS) of AMF community composition by place of sampling

From a total of 40 root samples collected in the Pond (Table 1), 17085 raw input sequences resulted from 454‐pyrosequencing, while 5596 sequences fulfilled the parameters of selection. A full reference maximum‐likelihood phylogenetic tree was used as “backbone” for the EPA approach and the placement of the query sequences (Figure S1 and Table S1), revealing 150 well‐defined AMF representative sequences from 2557 reads (i.e., from approx. 800 bp). The 150 AMF representative sequences were annotated as 15 species belonging to 7 genera (*Acaulospora*,* Archaeospora, Rhizophagus, Glomus, Sclerocystis, Dominikia* and *Kamienskia*) (Figure 2a,b). Some sequences could not be classified at the family or genus level. For instance, 5 OTUs were related either to *Rhizophagus* sp. or *Dominikia* sp. but could not be defined at the genus level and 1 OTU was in an undefined AMF branch from the reference phylogenetic tree (Figure 2a,b).

The genus *Archaeospora* was represented by two undefined species, one of them, *Archaeospora* sp. 1 was recorded only inside the Pond while *Archaeospora* sp. 2 was found in the three sampling places, with a lower abundance in the center of the Pond (Figure 2a). The genus *Acaulospora,* was represented by 6 species, *Acaulospora kentinensis, Acaulospora scrobiculata* and 4 *Acaulospora* spp. *Acaulospora kentinensis*, and 2 *Acaulospora* spp. (i.e., 2 and 4) were recorded in all the sites. *Acaulospora* sp. 3 was registered inside and in the center of the Pond, while *A. scrobiculata* and *Acaulospora* sp. 1 were registered only inside the Pond (Figure 2a). The genus *Rhizophagus* was represented by *R. proliferus* detected in the three sites and *Rhizophagus* sp. detected only inside the Pond. Additionally, 64 sequences (2.5% of relative abundance) detected in the center of the Pond were classified as either *Rhizophagus* sp. or *Dominikia* sp. *Glomus macrocarpum* was present inside and in the center of the Pond. The genus *Kamienskia* and *Sclerocystis* were represented by one undefined species and were detected only inside the Pond (Figure 2a). In contrast*,* only one OTU represented by 3 reads could not be classified at the family level, representing <1% of the species identified (Figure 2b) detected only inside the pond**.**

The NMDS analysis showed differences in the AMF community composition presented in the three sampling sites (Figure 2c). While the Shannon diversity index presented similar indices regardless of the sampling place (i.e., inside, outside, or center of the Pond with values of 1.60, 1.09, and 1.38, respectively).

## 4 DISCUSSION

Arbuscular mycorrhizal fungi are obligate roots symbionts associated with an approximate of 72% of vascular plants (Brundrett & Tedersoo, 2018) and are occurring in almost every ecosystem (Cabello, 2001). However, their presence in hydrocarbon‐polluted soils is poorly reported, though they may be of interest for remediation strategies (De la Providencia, Stefani, Labridy, St‐Arnaud, & Hijri, 2015; Lenoir, Fontaine, & Lounès‐Hadj Sahraoui, 2016; Lenoir, Lounes‐Hadj Sahraoui, Fontaine, 2016; Rajtor & Piotrowska‐Seget, 2016). Twenty‐five plant species (in 40 samples) were collected within the weathered oil crude Pond located in the Charapa field from the Amazon Basin of Ecuador. Root colonization by AMF was observed in all the plants. Total root colonization was above 40% and was very similar to a previous study (Garcés‐Ruiz et al., 2017). High colonization was also reported by Huang, Tang, Niu, and Zhang (2007) in 13 plant species growing in a petroleum‐contaminated soil at the Sichuan Province in China. Both results suggested that plants and AMF coexist under highly hydrocarbon‐polluted soil conditions.

However, 454‐pyrosequencing of an ~800 bp LSU rDNA fragment and a high‐throughput phylogenetic annotation method (EPA) were used in this study due to their robustness in short sequence characterization to species from environmental samples (Senés‐Guerrero & Schüßler, 2016a). This approach revealed a community composition of 15 AMF species composed of 1 *Glomus macrocarpum*–like species*,* 2 *Rhizophagus* spp., 6 *Acaulospora* spp., 2 *Archaeospora* spp. 1 *Sclerocystis* sp., 1 *Kamienskia* sp. One species was not attributed to a specific genus thus, leaving it in‐between *Dominikia* sp. and *Rhizophagus* sp. and one last representative sequence (RS) was determined as unclassified AMF. Interestingly, only the four‐first genera (*Glomus, Rhizophagus*,* Acaulospora* and *Archaeospora*) were detected by Sanger sequencing in Garcés‐Ruiz et al. (2017) in the same study site. However, in the study of Garcés‐Ruiz et al. (2017), only three plant species (representing 9 samples) were analyzed. The higher number of genera/species detected in this study could thus be attributed, at least partly, to the greater number of sequences generated from the 25 plants species (40 samples) analyzed.

*Acaulospora* was the more frequent genera (i.e., 73%) registered in the Pond. This prevalence was confirmed by the presence of *A. ketinensis*,* A. scrobiculata* and four unidentified species. Conversely, in the study of Garcés‐Ruiz et al. (2017), this genus was less abundant (13%) with the presence of *A. longula* associated with *Carludovica palmata* and one unindentified species associated with *Costus scaber* inside the Pond, while *A. ketinensis* was revealed outside the Pond colonizing *C. palmata* (Garcés‐Ruiz et al., 2017). The study performed by Iffis, St‐Arnaud, and Hijri (2016) also identified *Acaulospora* as a dominant genus in hydrocarbon‐polluted soils. Our study as well as the previous one (Garcés‐Ruiz et al., 2017) demonstrated thus the prevalence of this genus at different abundances. *Acaulospora* has been classified as stress‐tolerant (Chagnon, Bradley, Maherali, & Klironomos, 2013). Indeed, it was frequently reported under harsh climatic conditions in acidic soils (i.e., pH 3.6–4.20 (Morton, 2017)) as well as under high elevation sites such as the Andes region 2500 m asl (Loján et al., 2017). In our study, this genus was observed in alkaline soils (pH 8) as well as at lower pH (5–6) at 300 m asl, in highly hydrocarbon‐polluted conditions, demonstrating its ability to develop under highly contrasting conditions.

*Archaeospora* was represented by two unidentified species. The relative abundance of this genus was 19.6%. The abundance of this genus was different to the results obtained by Garcés‐Ruiz et al. (2017). *Archaeospora* was detected in the same Pond associated only with *C. scaber* with an abundance of ~50% and represented by one unidentified species. A Blast analysis between *Archaeospora* sp. sequences previously obtained from Sanger sequencing (Garcés‐Ruiz et al., 2017) and the RS from 454‐sequencing revealed a 95%–97% of shared identity (data not shown). Thus, we may hypothesize that the same unidentified species was detected in both studies.

Finally, the family Glomeraceae was represented by several species, although their relative abundance only represented 7% of the total AMF community. This could possibly be attributed to a lower affinity with the plant species sampled from the Pond or to an AMF species competition within the hydrocarbon‐polluted soil.

The AMF species that were not detected by Sanger but by pyrosequencing were *Glomus macrocarpum‐like, Sclerocystis* sp., *Kamienskia* sp., and *Dominikia* sp. The last two genera were only recently described (Błaszkowski, Chwat, Góralska, Ryszka, & Kovács, 2015). These two genera were identified by their spores, and it is thus suggested that their low occurrence may be attributed to a rare or seasonally sporulation or to their delicate spores easily decomposed by other organisms (Błaszkowski, Tadych, & Madej, 2000; Błaszkowski et al., 2015; Stutz & Morton, 1996).

*Sclerocystis* sp. and *G. macrocarpum*‐like were found at low occurrence. Conversely, in Garcés‐Ruiz et al. (2017), *Glomus* sp. was detected in *E. precatoria* and *C. palmata*. Its abundance was around 30%, while our results showed a relative abundance of 1.8%. Moreover, two OTUs of *Rhizophagus* sp. were closely related to *Sclerocystis sinuosa* (Garcés‐Ruiz et al., 2017), thereby our results by pyrosequencing confirmed its presence in the hydrocarbon‐polluted Pond.

The presence of *Rhizophagus proliferus* and a number of other undescribed *Rhizophagus* species demonstrated their ability to inhabit in soils containing high levels of TPH. This was not detected by Garcés‐Ruiz et al. (2017) with the Sanger sequencing method. In their study, this genus was only detected in the low‐hydrocarbon contaminated surrounding soil associated with *E. precatoria*. Although we could not precise to which plant species it was associated, it is suggested the adaptability of this genus to highly oil‐polluted soil as already revealed by Hassan et al. (2014), De la Providencia et al. (2015) and Iffis et al. (2016).

Unidentified AMF species accounted for 75% of the RS detected in our study. This corroborates the study of Garcés‐Ruiz et al. (2017) conducted in the same location, but using Sanger sequencing. Błaszkowski et al. (2015), suggested that only 5% of AMF species are identified to date based on phylogenetic analyses of sequences of nrDNA extracted from plant roots. Hence, it is not excluded that under highly perturbed and unexplored environments, the number of undescribed species may be even higher.

Within this study, a larger number of different AMF species were detected due to the greater number of plants collected and sequences generated as compared to the previous study of Garcés‐Ruiz et al. (2017). Our results improved the characterization of the AMF diversity capable to inhabit soils and colonize plant roots under highly polluted conditions in the Amazon region of Ecuador. The identification and characterization of undescribed species adapted to hydrocarbon pollutants could help in the selection of AMF species, production of inoculum, and its possible application for phytoremediation strategies.

## AUTHOR’S CONTRIBUTION

MG‐R: sampling, development of samples analysis, data collection, data analysis, interpretation of data. Drafting the work, commentaries corrections, final approval and agreement with all aspects of the work. CS‐G: contribution to the bioinformatics analyses and interpretation of the data, draft correction and final approval and agreement with all aspects of the work. SD: contributions to analysis of the results, draft corrections final approval and agreement with all aspects of the work. SC: substantial contributions from conception to data analysis, draft correction and final approval and agreement with all aspects of the work.

## CONFLICT OF INTEREST

We have no conflicts of interest to declare. Permits were given by the Ministry of environment of Ecuador and public enterprise PetroAmazonas EP for sampling and field study. The field study did not involve endangered or protected species.

## Data Availability

The data used in this manuscript are available at request from Laboratory of Mycology, Earth and Life Institute, Université catholique de Louvain, Louvain‐la‐Neuve, Belgium.
