# Supplementary figures and images for: Community composition of arbuscular mycorrhizal fungi associated with native plants growing in a petroleum‐polluted soil of the Amazon region of Ecuador
Source: Microbiologyopen. 2018 Aug 16;8(4):e00703. doi: 10.1002/mbo3.703 (PMC6529925; doi:10.1002/mbo3.703)

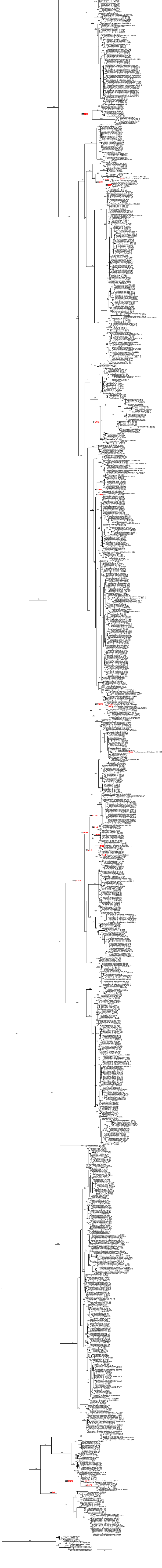

Supplement: Supplementary file 1 [file MBO3-8-e00703-s001.pdf]
